# A Case of Variegate Porphyria in Association With Coeliac Disease and Bisphosphonate Associated Dental Osteonecrosis

**DOI:** 10.4021/jocmr2009.11.1270

**Published:** 2009-12-28

**Authors:** Olayinka A. Ogundipe

**Affiliations:** a Department of Medicine of the Elderly, Royal Infirmary of Edinburgh, Edinburgh, EH16 4SA, United Kingdom. Email: ola_ayodele@hotmail.com

## Abstract

This case describes an older patient with a rare diagnosis of variegate porphyria presenting with acute abdominal pains and bloating, intermittent loose stools and jaw pains following surgical repair of an osteoporotic hip fracture. She was noted to have acute hyponatraemia. All the abdominal symptoms and the hyponatraemia were initially attributed to an acute episode of variegate porphyria with an accompanying syndrome of inappropriate antidiuretic hormone secretion. However, following further evaluations necessitated by the incomplete resolution of the abdominal symptoms, it became apparent that some of the persisting symptoms were due to a concurrent and new presentation of serology positive coeliac disease. The jaw pains were established to be due to dental osteonecrosis in association with the use of bisphosphonate therapy for treatment of osteoporosis. The various symptoms and signs subsequently settled uneventfully following institution of appropriate management options for the various coexisting diagnoses.

**Keywords:**

Abdominal pain; Abdominal bloating; Loose stools; Hyponatraemia; Variegate porphyria; Coeliac disease; Osteoporosis; Bisphosphonates; Osteonecrosis

## Case Report

A 73 years old caucasian woman of Scottish descent was admitted to hospital following a fall that resulted in a left femoral neck fracture. Her medical history was notable for variegate porphyria, hypothyroidism, chronic atrial fibrillation, rheumatoid arthritis and renal tubular acidosis. Medications on admission included paracetamol, omeprazole, levothyroxine, digoxin, warfarin and oral sodium bicarbonate supplements.

She had been previously diagnosed with variegate porphyria in her forties based on clinical features and laboratory tests. The diagnosis followed relevant urinary and faecal samplings during a presentation to hospital with acute abdominal symptoms and vesicular cutaneous eruptions to the sun-exposed areas of both upper extremities. There was also increased facial pigmentation. She had remained relatively symptom free since then, and had adhered conscientiously to the general counselling advice given (e.g. avoidance of precipitants of acute porphyria crises like multiple itemised medications, avoidance of low calorie diets, and avoidance of excessive exposure to sunlight).

Surgical repair of the hip fracture was performed with a dynamic hip screw (DHS). The immediate postoperative period was unremarkable. However, she subsequently developed a complication with low grade chronic infection to the DHS site. This required a period of antibiotics and the DHS thereafter required surgical revision to a gamma nail. Following the orthopaedic interventions she continued with a period of multidisciplinary rehabilitation. Multidisciplinary falls risk and osteoporosis assessments were completed and oral calcium/vitamin D supplements with weekly oral alendronic acid were prescribed.

Some six weeks post-operatively she developed acute abdominal pains. This was associated with abdominal bloating and intermittent loose stools of a non-infective nature. She also reported the onset of pains in the right maxillary region.

An acute-onset hyponatraemia developed and progressively worsened to a nadir of serum sodium concentration 116 mmol/L (normal 135 - 145). The evolving symptoms included anorexia, lethargy and gait imbalance. Mild peripheral oedema was noted. Paired serum and urine osmolalities were 245 mOsm/Kg (normal 280 - 296) and 301 mOsm/Kg respectively. Urinary sodium level was high at 89 mmol/L. She had normal serum cortisol responses on short synacthen testing. Other relevant evaluations including serum potassium, calcium, magnesium, triglycerides, serum protein electrophoresis, urine Bence-Jones protein screening, and chest X-ray were unremarkable. She had a mild dimorphic anaemia due to a combination of iron and folic acid deficiencies. Serum vitamin B12 level was normal. Serum digoxin levels were within the normal range and thyroid function tests suggested optimal supplementation. Serum renal function tests were unremarkable with an estimated glomerular filtration rate (eGFR) of over 60 ml/hr.

A general surgical review supported by abdominal computerised tomography scanning did not identify any acute surgical cause for the abdominal pains. A dental review established that the jaw pains were due to dental osteonecrosis involving the right maxillary bone. The symptoms of the osteonecrosis gradually responded to conservative management involving discontinuation of the alendronic acid, oral and dental hygiene measures, oral antibiotics for secondary infection, and analgesia. A medical review attributed the acute abdominal symptoms and acute onset hyponatraemia to an acute episode of variegate porphyria.

She showed some symptomatic improvement with the institution of a cautious regimen of fluid restriction as initial management for possible SIADH-associated hyponatraemia; and a high calorie diet as initial management for the suspected acute porphyria episode. The nutritional management was supervised by a dietician. A period of supplemental nasogastric (NG) tube feeding was required, aimed at providing a high calorie carbohydrate intake, and whilst striving to maintain an initial restricted fluid intake target of 1 litre in 24 hours. On this management regimen, the serum sodium concentration normalised to 138 mmol/L, the abdominal pains resolved and there was marked improvement in the lethargy and gait imbalance. The supplemental NG feeding was discontinued when her appetite improved to the point of allowing satisfactory oral food intake. The fluid restriction was progressively relaxed to 1.5 litres in 24 hours, and subsequently to 2 litres in 24 hours without recurrence of the hyponatraemia.

Despite the initial clinical and biochemical improvements described above, she reported persistence of intermittent abdominal bloating and recurring bouts of loose stools. Multiple stool samples for routine microscopy, cultures and clostridium difficile toxin screens returned clear. These persistent features, along with the presence of the dimorphic anaemia, informed the subsequent decision to screen her for coeliac disease.

Her IgA tissue transglutaminase antibody (tTG-IgA) levels returned markedly elevated at 125 AU (normal 0.1 - 7.9 AU). She received appropriate counselling towards undertaking an upper gastrointestinal endoscopy (UGIE), to allow for the (gold-standard) histological confirmation of the diagnosis of coeliac disease from distal duodenal biopsies. However, she declined an UGIE citing physical frailty and all the recently endured medical hardships. Following further counselling she consented to an empirical trial of a gluten-free diet (GFD) on the clinical suspicion of possible serology positive coeliac disease.

The GFD was cautiously introduced under the close supervision of a dietician so as to avoid a sudden reduction to her calorie requirements and the risk of precipitating another episode of acute porphyria. Following establishment on the GFD diet for about three weeks and discontinuation of all fluid restrictions, the residual abdominal symptoms of bloating and loose stools resolved. The serum sodium and other relevant serum biochemistry remained normal. Of note however was a marked urinary sodium loss that persisted on a pre-discharge sample (urinary sodium 159 mmol/L).

She thereafter progressed well with a further period of multidisciplinary rehabilitation, regaining functional independence with her transfer abilities and with mobility supported with the use of a wheeled zimmer frame as a physical aid. Modified cutlery assisted her in overcoming the restrictions of rheumatoid arthritis to both hands. Minor assistance was required with washing and dressing, and she was discharged home with her family providing informal assistance with care.

Following discharge from hospital she has remained symptomatically well on the GFD, with repeat levels of her tTG-IGA levels showing reducing titres to 70.7 AU (four months following commencement of a GFD).

## Discussion

### Porphyria

The porphyrias are a rare group of medical conditions characterised by abnormalities in the haem biosynthetic pathway [1]. Most cases are inherited (e.g. acute intermittent porphyria, hereditary coproporphyria and variegate porphyria), whereas some cases may be an acquired disorder (e.g. porphyria cutanea tarda). The porphyrias can be classified further according to the predominant source of overproduction of porphyrias and precursors of porphyrin (e.g. hepatic or bone marrow), or based on the predominant clinical presentation (neurovisceral or cutaneous) [1-6].

Variegate or mixed porphyria (VP) is considered to be a hereditary disorder which is characterised by a reduction in the hepatic protoporphyrinogen oxidase enzyme of the haem synthetic pathway (at the stage of oxidative conversion of protoporphyrinogen IX to form protoporphyrin IX). VP can remain asymptomatic for long periods, but during an acute episode individuals may present with neurovisceral and/or cutaneous symptoms or signs, some of which may be serious or even life-critical [1].

Cases of VP are described rarely in the medical literature. Often, these cases describe the practical difficulties of establishing a firm diagnosis, and the various added problems in managing cases when they present with acute exacerbations [1-10]. Hyponatraemia and abdominal pains are well recognised manifestations of the acute porphyrias and can occur irrespective of the subtype. Nevertheless, consideration of the porphyrias as a possible diagnosis in routine practise can still be easily overlooked because of the rarity, and because hyponatraemia and abdominal pains are fairly common presentations that can occur in commoner differential diagnoses.

For first presentations in patients without a known or pre-existing diagnosis of porphyria, it has been suggested that the pragmatic emphasis of the acute period of care should be a focus on establishing the diagnosis of an acute porphyria, and the early institution of standard therapeutic options. As the acute treatment strategies generally overlap, it may be prudent to concentrate the focus of initial management on the generic treatments that may reduce morbidity and mortality, and not to defer treatment on the basis of ongoing efforts to accurately subtype the porphyria, a diagnostic process that often continues into the post-acute phase and that is usually informed by evolving clinical and laboratory evaluations [1].

In the index case, it is hypothesised that stressful triggers (independently or in combination) such as the preceding major surgeries, anaesthesia, infection, reduced calorie intake in the post-operative phase, medications, and/or pain may have variably contributed to the precipitation of an acute onset of porphyria symptoms/signs in an individual with known VP, but with a preceding prolonged symptom free phase of about thirty years.

The mechanism of the hyponatraemia seen in acute porphyria is not certain but postulates include a link to the syndrome of inappropriate antidiuretic hormone secretion (SIADH), and a resultant renal tubular retention of free water. This patient had serum and urine osmolalities, and elevated urine sodium levels that would be supportive of SIADH. However, according to strict diagnostic specifications, one could not state categorically that the hyponatraemia in this case was due to SIADH because of the diagnostic restrictions/exclusions that could apply e.g. a requirement for euvolaemia, normal thyroid function, and normal renal function amongst others, as well as the fact that the patient was taking oral sodium bicarbonate tablets for previously diagnosed renal tubular acidosis.

The use of oral demeclocycline as a therapeutic option to manage the acute, severe and symptomatic hyponatraemia was considered in this case. Unfortunately, use of this medication is specifically contraindicated in porphyria. The option of using intravenous (IV) haematin was also considered. However, in view of the fairly good initial response noted to the fluid restriction and dietary (calorie) modifications, the option of IV haematin was reserved as a second line intervention. A decision was made to use IV haematin if she showed signs of clinical deterioration, but as her clinical and biochemical picture showed continuous improvement she did not require this.

As some cases of VP may be inherited as an autosomal dominant trait, the patients grownup children were referred to a tertiary clinical genetics department for consideration of elective genetic screening, subject to the granting of consent after appropriate counselling. Genetic screening is currently possible to detect the presence of a splice site mutation in the gene for protoporphyrinogen oxidase; a finding that would be consistent with a diagnosis of VP [1].

### Coeliac disease

Coeliac disease (CD) is an autoimmune condition that is probably under-recognised and under-diagnosed [11]. UK based studies have described a prevalence rate ranging between 0.9 - 1.8% [11]. It is now acknowledged that CD has the potential to present variably in children, adults or older individuals [11-13].

The gastrointestinal features of CD generally have greater recognition (e.g. chronic or intermittent diarrhoea; persistent or recurring and unexplained nausea and vomiting; recurrent abdominal cramps, pain or distension/bloating; weight loss; and unexplained iron deficiency anaemia or dimorphic anaemia) [11].

However, the non-gastrointestinal associations of CD are also being increasingly described and recognised (e.g. osteoporosis, osteomalacia, an increased risk of intestinal lymphoma, epilepsy/seizures, etc), as are its associations with other autoimmune diseases (type 1 diabetes, autoimmune thyroid disease, Addisons disease, etc) [11-14].

Individuals with IgA deficiency may return false-negative IgA-based serological tests (e.g. tissue transglutaminase and endomysial antibodies). In these cases, IgG-based serological tests may be required as part of the diagnostic evaluations. Although not applicable to this patient, false positive tissue transglutaminase antibody results have been described in individuals with chronic liver disease, myeloma, monoclonal gammopathy, and type 1 diabetes [11].

Regardless of a positive IgA-tTG serology which is suggestive of possible CD, as is the case in this report, it is important to emphasise that current guidelines recommend histological diagnosis on small intestinal biopsy as being essential before a definitive diagnosis of CD can be made, and before a GFD would generally be instituted [11,15]. However, as mentioned earlier the patient in this case had a strongly positive serology screen but declined the option of UGIE after counselling. She however granted consent to a trial of a GFD on the clinical suspicion of serology positive CD. As the GFD has translated to symptomatic benefit she has elected to continue on this.

### Bisphosphonate associated dental osteonecrosis

Bisphosphonates are a recognised and evidence-based management option for osteoporosis and generally feature high up in treatment algorithms. Bisphosphonate associated osteonecrosis has previously been described as a relatively uncommon condition when its incidence is compared to its general use in clinical practise. However, the increasing use of bisphosphonates variably for the management of osteoporosis, hypercalcaemia, bones pains due to metastatic disease or Pagets disease etc, may with time translate into an increasing incidence of reports of osteonecrosis. It is noteworthy that although not isolated to such cases, the trend in the reporting of cases has leaned more towards bones around the jaw/mouth, those in which high dose bisphosphonates have been employed, those on prolonged therapy (in many cases greater than one year), and particularly when therapy has involved administration through the intravenous route [16-18].

The patient in this report was receiving oral alendronic acid (a bisphosphonate) as part of an individualised management plan for osteoporosis and had been on treatment for only a few months. On discontinuation of the alendronic acid because of the known association with osteonecrosis, the possibility of prescribing alternatives for the osteoporosis was explored e.g. oral strontium ranelate etc. Unfortunately, experience and formulary information on the use of strontium ranelate or other alternative osteoporosis treatments in porphyria is limited. This appears to be largely as a result of the rarity of the porphyrias.

Theoretical arguments would suggest that as strontium ranelate is not metabolised by and does not inhibit the hepatic cytochrome P450 enzyme, there should be no problems if administered to individuals with VP, the latter itself being an example of a hepatic porphyria. However, in this case the patient declined the option of taking strontium ranelate given the lack of clinical experience with its use in porphyria.

### Further thoughts and conclusion

A search of the medical literature produced a solitary report of a case of variegate porphyria in an individual with coeliac disease. The patient also had beta-thalassaemia minor [7]. The current case represents another rare report of variegate porphyria in an individual with serology positive coeliac disease.

The medical literature identifies CD as a risk factor for osteoporosis with an accompanying association with increased fracture risk [19,20]. Other risk factors for osteoporosis in this case include a postmenopausal status and chronic rheumatoid arthritis. This patient developed dental osteonecrosis in association with use of an oral bisphosphonate that was prescribed for the treatment of osteoporosis. Though by no means proven, it is plausible that dental enamel deficiencies may have played a further predisposing role in this case, considering that the medical literature also proposes that CD may be associated with dental enamel defects [11,12].

Abdominal complaints are common, and in some instances may be subsequently identified as being due to initial presentations of CD, or less commonly due to acute porphyria. This report serves as a reminder for clinicians to maintain a degree of diagnostic openness when symptoms/signs that initially appear clear-cut do not appear to improve or resolve satisfactorily upon institution of initial treatment options for a primary working diagnosis. This is especially important when the primary diagnosis is in itself rare.

In medicine, the term sequence is sometimes used in reference to a series of consequences arising in an ordered manner from a single cause. In this case, it is possible that coeliac disease could have predisposed to osteoporosis, and the bisphosphonate that was duly prescribed for osteoporosis predisposed to dental osteonecrosis. The index case is the first describing an individual with variegate porphyria, possible coeliac disease and bisphosphonate associated osteonecrosis.

Other notable associations in this case include the presence of the possible SIADH, renal tubular acidosis (type 2 / proximal tubular), hypothyroidism and rheumatoid arthritis. Renal tubular acidosis is itself associated with osteomalacia and proximal myopathy; with muscle weakness being recognised as a risk factor for increased risks of falls. A predisposition to falls increases the risk of fractures, particularly in individuals with co-existent osteoporosis.

The presence of many of these varied conditions (variegate porphyria, coeliac disease, hypothyroidism, renal tubular acidosis and rheumatoid arthritis) in a single individual raises a question regarding the possibility of genetic associations or influences intertwining some or all of them. This is an area that future research may want to explore and offer confirmatory or refuting evidence.

### Key points

1. Variegate porphyria is a rare condition and an acute exacerbation can present with abdominal pains and acute hyponatraemia.
2. Management options for acute porphyria include avoidance of precipitating medications or stressors, provision of high calorie diets or intravenous glucose supplementation, and intravenous haematin.
3. A rare association between porphyria and coeliac disease may mask or confuse the clinical picture in individuals presenting with abdominal symptoms.
4. Coeliac disease can present newly even in older patients with iron deficiency anaemia, weight loss, or with abdominal symptoms like bloating, cramps and loose bowel motions.
5. Management options for coeliac disease include a gluten free diet under supervision and usually supported with histological monitoring of response.
6. The place of serological monitoring of response in coeliac disease has not been clearly established, particularly in patients that decline, or are unable to undergo upper gastrointestinal endoscopies on an initial diagnosis or response monitoring basis.
7. Coeliac disease is a risk factor for osteoporosis and dental enamel defects.
8. Bisphosphonates prescribed for the management of osteoporosis can predispose to osteonecrosis, with the jaw bone regions appearing especially susceptible.
9. Genetic screening following counselling for families is now a valuable option in the diagnostic armoury for further evaluation of suspected index cases or in the blood relatives of individuals with porphyrias. Genetic testing has the added advantage that tests can be performed even in asymptomatic blood relatives; a scenario in which standard urine and faecal samplings may prove significantly less rewarding.
